# Predictors of self-reported health among the elderly in Ghana: a cross sectional study

**DOI:** 10.1186/s12877-017-0560-y

**Published:** 2017-07-31

**Authors:** Cynthia Lum Fonta, Justice Nonvignon, Moses Aikins, Emmanuel Nwosu, Genevieve Cecilia Aryeetey

**Affiliations:** 1 Department of Health Policy Planning and Management, School of Public Health, University of Ghana, Legon, Accra LG 13 Ghana; 2 Department of Economics, University of Nigeria Nsukka, Enugu State, Nigeria; 3 West African Science Service Center on Climate Change and Adapted Land Use, WASCAL Competent Center, Blvd Mouammar Kadhafi, 06, Ouagadougou, BP 9507 Burkina Faso

**Keywords:** Self-reported health, Elderly population, Wellbeing, Ghana

## Abstract

**Background:**

Self-reported health is a widely used measure of health status across individuals. As the ageing population increases, the health of the elderly also becomes of growing concern. The elderly go through life facing social, economic and financial hardships. These hardships are known to affect the health status of people as they age. The purpose of this study is to assess social and health related factors of self-reported health among the elderly in Ghana.

**Methods:**

A multivariate regression analysis in form of a binary and ordinal logistic regression were used to determine the association between socioeconomic, demographic and health related factors, on self-reported health. The data used for this study was drawn from the World Health Organization (WHO) Study on Global Ageing and Adult Health (SAGE) Wave 1.

**Results:**

In total, out of 2613 respondent, 579 (20.1%) rated their health status as poor and 2034 (79.9%) as good. The results showed that the odds of reporting poor health was 2.5 times higher among the old-old compared to the young old. The elderly with one or more than one chronic condition had the odds of 1.6 times and 2 times respectively, of reporting poor health. Engaging in mild to moderate exercise increased the chances of reporting poor health by 1.8 times. The elderly who had never worked in a lifetime were 2 times more likely to report poor health. In the same way, residents of Eastern and Western parts of Ghana were 2 times more likely to report poor health compared to those in the Upper West region. Respondents with functional limitations and disabilities were 3.6 times and 2.4 times respectively, more likely to report poor health. On the other hand, the odds of reporting poor health was 29, 36 and 27% less among respondents in the highest income quintiles, former users of tobacco and those satisfied with certain aspects of life respectively. Also, current alcohol users were 41% less likely to report poor health.

**Conclusion:**

The health status of the elderly is to an extent determined by the circumstances in which they are born, grow and live. The findings suggest that addressing social issues faced by individuals in youthful age will go a long way to achieving good health in the future. People with physical limitations and disabilities are most vulnerable to unmet healthcare needs and support system from government, policy makers and family.

## Background

Self-reported health (SRH) has been used by many researchers as a proxy for measuring individual health state, including that of the elderly [1–3]. It not only measures current and long term health status but also predicts mortality [4]. Self-reported health which comprises of all three aspects of health: social, mental and physical wellbeing has been widely studied in the western world using different methods. Most findings show a significant relationship between certain social, economic and demographic factors and SRH. It is important to examine past life experiences of elderly persons together with individual characteristics when exploring differences in reporting health. Some of these experiences include cultural factors, historical (wars), individual and national factors (availability of health services) [5]. In North Korea, for example, among North Korean refuges living in South Korea, Wang et al. found poor SRH to be related to individuals of low socioeconomic statues, elderly females and those with physical and mental disabilities including other chronic conditions [6], owing to past wartime experiences and economic recession. Other studies have found that individual factors like low educational attainment increases the chances of reporting poor health [7, 8]. A possible pathway could be through lack of employment, low economic condition and consequential inability to take care of healthcare needs. In the same way, socio-cultural factors have an impact on SRH across racial-ethnic groups [9] owing to the different perception of health. To be precise, objective health states such as presence of chronic condition is positively associated with poor health among the less racially discriminated blacks, while demographic factors are highly associated with SRH among the most racially discriminated African Americans.

Evidence have also shown that being single or divorced increases the chance of reporting poor health [10]. This is often associated with emotional instability, loneliness as well as economic vulnerability. In the same light, low income, poor lifestyle choices such as smoking, excessive drinking and lack of exercise are other risk factors associated with poor health [11]. Smoking for example increases the chances of dying from medical conditions like ischemic heart disease, lung cancer, stroke and coronary heart disease [12, 13]. Sedentary lifestyle can predispose one to these diseases as well. However, research shows that physical activity in the form of exercise has the potential of reducing the onset of chronic disease and thus improve health outcome [14, 15]. Some of these health consequences are reversible when the individual is no longer exposed to these poor habits. In terms of physical health states, there is a growing body of evidence that relates functional limitation and disabilities to poor health [16–18]. Activities of Daily Living (ADL) and Instrumental Activities of Daily Living (IADL) are used globally to measure functional health states of adults [19, 20]. These health states, IADL often present in states of mild cognitive impairment or early dementia [21] whereas ADL declines present in the late stages of dementia [22]. Exploring what drives SRH among elderly persons will provide baseline evidence to uphold public health promotive and preventive strategies against disease onset. Some school of thought hold that SRH can be better understood when an attempt is made to explore the rationale behind the responses in order to illustrate the sociocultural context of how individuals assess their health state [23].

The population of the world is ageing. The number of people aged 60 years and older have been projected to increase from its current state of 800 million to two billion by the year 2050 [24]. In Ghana for example, the population of the elderly (ages 60 years and above) is expected to double from its current state of 6.7% of the national population to more than 12% by the year 2050 [25]. Indeed, the age structure of Ghana’s population is gradually changing following the global pattern. This can be attributed to the decline in mortality and fertility rates and an increase in life expectancy, from advancement in medical technology and improved living standards [26]. Concerning elderly health, few studies exist on the elderly with respect to SRH and risk factors in Ghana. One of such health related risk factor among elderly Ghanaians is the presence of chronic diseases. The most common of them include cardiovascular diseases, cancers, respiratory diseases, arthritis and other infectious diseases [27]. Other health challenges include chronic malnutrition, anemia, osteoporosis, hearing and sight problems. In general, causes of chronic conditions are multifaceted ranging from genetic predisposition, poor lifestyle decisions to poor social and economic conditions, all these having as consequence, a negative impact on elderly health status [28, 29]. With the rising prevalence of chronic diseases, the health system in Ghana is less prepared to meet the health care needs of the elderly, with little infrastructure and few specialized personnel for older populations [30]. Further, in terms of health access, Saeed et al. found that most elderly persons in Ghana reporting poor health use health facilities more owing to the National Health Insurance policy which offers free healthcare to elderly persons above 70 years [31]. While this is seen as a positive development at national level, the quality of services rendered are not encouraging and the range of services offered are limited.

Although elderly women in Ghana have longer life expectancy than men, they report worst health outcomes compared to men [32]. This is in line with other studies that have shown gender difference in reporting poor health [33, 34]. Also among elderly Ghanaians, Debpuur et al. [35] found SRH to be associated with education, functional limitation and lifetime employment. However, this single study on SRH in Ghana used primary data from one rural district, which is not representative of the national situation. Given variations in health status across locality, this study aims to assess the factors that influence the health status of the elderly, with self-reported health as a measure of health outcome using a national-level dataset. It is worth noting that most interventions in Ghana focus on children and youthful population, with little emphasis on the elderly. Understanding what explains SRH among elderly persons in Ghana is thus a first step to improving their health state through successful implementation of policies, interventions and programs.

## Methods

### The aim, design and setting of the study

The aim of the study was to assess factors associated with SRH among the elderly in Ghana. The study used secondary data obtained from a cross national Study of Global Ageing and Adult health (SAGE) Wave 1, a cross sectional study conducted in Ghana from 2007 to 2010 by the World Health Organization (WHO). The data was obtained from the WHO, publicly available online with no identifiable information on participants [36]. Prior to the study, 30 interviewers and supervisors were trained in two phases in Accra, supported by WHO Geneva. There were a total of four interviewers and a supervisor for each primary sampling unit. They interviewed at most two respondents per day and each interview lasted for at most 30 min [37]. For quality control measures, the supervisors checked to ensure that proxy interviews were justifiable and completed. In addition, selected variables in the questionnaires were re-tested a week after initial interview for consistencies.

The study sample was stratified by 10 administrative regions and then by locality into urban and rural areas. A total of 20 strata were identified. In the urban and rural areas, census enumerated areas were used as the sampling frame. A total of 251 census enumerated areas were identified. Enumerated areas were then selected from each stratum based on the size of the population of the elderly in that area. Twenty households were randomly selected from each enumerated area. Adults older than 50 years were selected from each household, and individuals between the ages of 18–24 years were selected as proxies. The analysis in this study defines the elderly as those aged 60 and above resulting in a sample of 2613 individuals. The data was collected by face to face interviewer administered questionnaires to each household and individual respondent. The questionnaires were divided into various sections that assessed the different characteristics of the elderly such as socio-demographic characteristics, work history and benefits, health state descriptions, chronic conditions and health services coverage.

### Description of materials

#### Dependent variable

The dependent variable used for this study was self- reported health. The survey contained the following questions as it relates to SRH: ‘How do you rate your health today?’ Responses were rated on a 5-point scale as ‘very good’ (1), ‘good’ (2), ‘fair’ (3), ‘poor’ (4), and ‘very poor’ (5). Responses were further classified into a dichotomous measure; ‘good health (very good/good/fair) defined as optimal health state and coded as 0, while poor health (/poor/very poor) as less than optimal health, coded as 1. This approach of dichotomizing the health variable was adopted from previous studies on self-reported health [38–40]. We used similar approach which include SRH as the dependent variable comprising of good health, 0 and poor health 1. Other approaches have been used by different scholars. For example, Arnadotti and colleagues analyzed all five health states of SRH using ordinal logistic regression [41] whereas Wu et al. used three SRH states good, fair and poor [42]. For the purpose of sensitivity, we used an ordinal logit model, using all five health states to have a broad perspectives of factors associated with SRH among elderly persons in Ghana. That is, SRH was coded as 5 when very poor, and 1–4 otherwise.

### Independent variables

The explanatory variables include demographic factors such as age, sex, marital status. Following the classification by the 2010 population census in Ghana, age was classified into three groups - the young old (60–69), the middle old (70–79) and the old-old (above 80) [43, 44]. In respect to marital status, respondents were classified as never married, married/cohabiting, separated/divorced and widowed. Socioeconomic factors included income, education and work status. Income was grouped into five quintiles (highest, high, middle, low and lowest). It was reclassified into three categories; high (high/highest), middle income (middle), low income (low/lowest). The SAGE study relied on income from household assets, dwellings characteristics and reported incomes which were converted into income quintiles, with quintile 1 representing the lowest income and quintile 5 representing the highest income. Education was grouped into (no formal education, primary education, secondary and tertiary education). Lifetime work status was grouped into four categories (never worked, private employer, public employer and informal). Health insurance status categorized into two groups, being insured and uninsured.

The classification of chronic diseases were based on the respondent’s response to the question ‘In the past 12 months, have you been told by a medical professional you had any of the following conditions (diabetes, hypertension, arthritis, angina, stroke, chronic lung disease, depression and asthma?’ Three categories were formed from the responses given, no chronic condition, one chronic condition and more than one chronic condition.

Functioning assessment was evaluated using the questions from SAGE questionnaire found under the section Health State Description. Functional limitation was measured using ADL. Respondents were asked if they had difficulties in carrying out any of the following eight activities (sitting, walking, standing from sitting, long periods of standing, climbing stairs, kneeling, picking up things and taking care of household responsibilities). Each of these 8 items were classified on a four point response scale. A dichotomy measure was constructed grouping no limitations in any of the activities as 0 and mild to severe limitations as 1. Respondents were considered functionally limited if they experienced difficulties in carrying out 4 or more of these activities. Disability was constructed based on the World Health Organization Disability Assessment Schedule (WHODAS 2.0) made up of 15 questions also referred to as IADL [35, 45]. Respondents were considered disabled if they had difficulties in performing 7 or more of these 15 activities [46].

In terms of subjective wellbeing, respondents were asked how satisfied they were with their health state, themselves, personal relationships, living conditions and in general life. Subjective wellbeing was seen to be good if respondents were satisfied with four or five of the domains and poor if satisfied with three or less. A self-reported response of how well respondents rated their quality of life was used to construct the quality of life variable. Responses were rated on a 5 point scale: ‘very good’ ‘good’ ‘fair’ ‘bad’ and ‘very bad.’ Very good and good responses were grouped as good quality of life, fair, bad and very bad as poor quality of life.

Lifestyle factors include use of any of the following tobacco products like cigarettes, cigars, pipe, chewing tobacco or snuff, alcohol use and physical activity. Respondents were grouped into three groups “never used”, “previously used” and “currently use” tobacco. Alcohol was grouped into three groups as well, “non-drinkers” of alcohol, “previous drinkers” and “current drinkers”. Physical activity was assessed on the basis of any form of moderate to intensity sports that increased the heart rate for at least 10 min a day.

Geographical characteristics include urban-rural locality and regions (Upper West, Western, Upper East, Central, Ashanti, Brong Ahafo, Northern, Eastern, Greater Accra and Volta). Upper West is considered the poorest region in the country hence was used as the base case for comparison with other regions.

### Statistical analysis

STATA version 13 was used as statistical package for data analysis. First, a descriptive analysis was done to describe the general characteristics of the study population. Second, we explored the difference in reporting SRH against the independent variables, reporting *p*-values of Pearson’s Chi^2.^ Third, a multivariate analysis in the form of a binary logistic regression and an ordinal regression analysis were used to determine the association between social/health related factors and self-reported health. Odd’s ratio (OR) was used as a measure of effect. The level of significance was set at a 95% confidence interval with a *p*-value of 0.05.

#### Background characteristics of respondents

Out of 2613 respondents, 20% reported poor health and 80% reported good health (Table 1). The young old (60–69 years) represented 45% of the sample, middle old (70–79 years) 38% and the old-old (80 and above), 17% of the entire population. Equal number of males (50%) and females (50%) were represented in the sample. In terms of marital status, most respondents were married (49%), followed by 37% of elderly persons who had lost a spouse. More than half (66%) elderly persons had never been to school and just a small proportion (3%) had completed tertiary education.

**Table 1:** Descriptive characteristics of the study population

Variables	Frequency (n)	Percent (%)
SRH		
Good health	2034	79.9
Poor health	579	20.1
Age		
60–69	1204	45.1
70–79	983	37.6
Above 80	426	16.3
Sex		
Male	1314	50.3
Female	1299	49.7
Marital Status		
Never married	27	1.0
Married/co-habiting	1268	48.8
Separated/divorced	351	13.5
Widowed	950	36.6
Education Level		
No formal education	1726	66.4
Primary	443	17.0
Secondary	359	13.8
Tertiary	73	2.8
Work status		
Never worked	47	1.8
Public	219	8.4
Private	82	3.2
Informal	2254	86.6
Health insurance		
Uninsured	1482	56.7
Insured	1131	43.3
Chronic condition		
Chronic condition	1665	63.9
One chronic condition	619	23.8
More than one condition	320	12.3
Income quintile		
Lowest	1106	42.4
Middle	530	20.3
High	973	37.3
Locality		
Urban	1012	38.7
Rural	1601	61.3
Subjective wellbeing		
Satisfied	894	34.21
Not satisfied	1719	65.79
Quality of life		
Good	1040	39.8
Poor	1573	60.2
Functional limitation		
Not functionally limited	1328	50.8
Functionally Limited	1285	49.2
Disability		
Not disabled	1622	62.1
Disabled	991	37.9
Physical activity		
None	1999	76.6
Moderate exercise	330	23.4
Tobacco Use		
Never used	2035	77.9
Previously used	283	10.8
Currently use	295	11.3
Alcohol		
Non-drinker	1243	47.6
Previous drinker	632	24.2
Current drinker	738	28.2
Region		
Upper West	273	2.9
Western	294	11.3
Central	281	10.8
Ashanti	390	14.9
Brong Ahafo	252	9.6
Northern	247	9.5
Upper East	174	6.7
Greater Accra	76	10.4
Eastern	360	13.8
Volta	266	10.2

Table 1 further shows that the highest proportion of respondents (87%) were employed in the informal sector, and just a few employees (8 and 3%) in the public and private sectors respectively. Fifty seven percent of elderly Ghanaians lack health insurance coverage while 43% had some form of insurance coverage. With respect to comorbidities, 24% elderly men and women had one of the eight chronic conditions explored in this study and 12% had more than one of these conditions. Forty two percent were considered poor and most (61%) lived in the rural areas. In terms of subjective well-being, 66% elderly persons were unsatisfied with life and their surroundings, 60% rated poor quality of life, 49% were functionally limited and 38% were classified as disabled.

Lifestyle factors assessed showed that the same proportion of elderly Ghanaians (78%) do not exercise or take any form of tobacco. In terms of drinking habits, 47% elderly men and women do not drink, 24% were previous drinkers and 27% current drinkers. Based on regions, the table shows that most (15%) elderly persons were from the Ashanti region, followed by the Eastern (14%) and Western (11.3%) regions respectively. Just a few proportions were from the Upper West (3%).

#### Summary statistic of the population by self-reported health

In comparing the differences in SRH across the various socioeconomic demographic and health related factors, the proportion of respondents reporting good health in all categories were more than those reporting poor health. This is represented in Table 2. Age was statistically related to SRH and the highest proportion (83%) of respondents reporting good health were the young old while the oldest old reported poor health more (36%). In other words, reporting good health declined from 83 to 64% as age increased while poor health increased with age, from 17 to 36%. Most males (84%) were seen to report good health compared to 71% of females. On the other hand, 26% of females reported poor health while only 18% of males reported poor health.

**Table 2:** Summary statistics by self-reported health

Variables	Good Health (%)	Poor Health (%)	Total (*N*)	*P*-values of Pearson’s Chi^2^
Age				<0.000
60–69	1000 (83.1)	204 (16.9)	1204	
70–79	762 (77.5)	221 (22.5)	983	
Above 80	272 (63.8)	154 (36.2)	426	
Sex				<0.000
Male	1057 (81. 4)	242 (18.6)	1299	
Female	977 (74.4)	337 (25.6)	1314	
Marital Status				<0.000
Never married	18 (66.7)	9 (33.3)	27	
Married/co-habiting	1034 (81.6)	234 (18.4)	1268	
Separated/divorced	269 (76.6)	82 (23.4)	351	
Widowed	699 (73.6)	251 (26.4)	950	
Education Level				<0.000
No formal education	1312 (76.0)	414 (24.0)	1726	
Primary	352 (79.5)	91 (20.5)	443	
Secondary	295 (82.2)	64 (17.8)	359	
Tertiary	65 (89.0)	08 (11.0)	73	
Work status				0.021
Never worked	31 (66)	16 (34.0)	47	
Public worker	182 (83.1)	37 (16.9)	219	
Private worker	69 (84.1)	13 (15.8)	82	
Informal worker	1742 (77.3)	512 (22.7)	2254	
Health insurance				0.482
Uninsured	1161 (78.3)	321 (21.7)	1482	
Insured	873 (77.2)	258 (22.8)	1131	
Chronic condition				<0.000
No chronic condition	1356 (81.4)	309 (18.6)	1665	
One chronic condition	448 (72.4)	171 (27.6)	619	
More than one condition	221 (69.1)	99 (30.9)	320	
Income				0.021
Lowest	840 (76.0)	266 (24.0)	1106	
Middle	405 (76.4)	125 (23.6)	530	
High	786 (80.78)	187 (19.22)	973	
Locality				0.791
Urban	785 (77.6)	227 (22.4)	1012	
Rural	1249 (78.0)	352 (22.0)	1601	
Subjective Wellbeing				0.131
Satisfied	709 (79.3)	285 (20.7)	894	
Not satisfied	1325 (77.1)	394 (22.9)	1719	
Quality of Life				0.111
Good	793 (76.3)	247 (23.7)	1040	
Poor	1241 (78.9)	332 (21.1)	1573	
Functional limitation				0.095
Not functionally limited	1016 (76.5)	312 (23.5)	1328	
Functionally limited	1018 (79.2)	267 (20.8)	1285	
Disability				0.133
Not disabled	1247 (76.9)	375 (23.1)	1622	
Disabled	787 (79.4)	204 (20.6)	991	
Physical activity				<0.000
None	1583 (79.2)	416 (20.8)	1999	
Moderate exercise	451 (73.5)	163 (26.5)	614	
Tobacco use				0.005
Never used	1574 (77.4)	461 (22.6)	2035	
Previously used	210 (74.2)	73 (25.8)	283	
Currently use	250 (84.7)	45 (15.3)	295	
Alcohol use				0.615
Non drinker	973 (78.3)	270 (21.7)	1243	
Previous drinker	483 (76.4)	149 (23.6)	632	
Current drinker	578 (78.3)	160 (21.7)	738	
Region				<0.000
Upper West	65 (85.5)	11 (14.5)	76	
Western	207 (70.4)	87 (30.0)	294	
Central	221 (78.6)	60 (21.4)	281	
Ashanti	301 (77.18)	89 (22.8)	390	
Brong Ahafo	199 (79.0)	53 (21.0)	252	
Northern	205 (83.0)	42 (17.0)	247	
Upper East	158 (90.80)	16 (9.20)	174	
Greater Accra	215 (78.8)	58 (21.2)	273	
Eastern	263 (73.1)	97 (26.9)	360	
Volta	200 (75.2)	66 (24.8)	266	

A high proportion of elderly persons (82%) who were married reported good health while poor health was reported most (26%) by the widowed. Most elderly Ghanaians with tertiary education (86%) reported good health and just a few proportion (11%) reported poor health. Respondents in the private sector reported good health the most (84%) while those who had never worked reported the highest proportion of poor health (34%). Eighty one percent of respondents with none of the listed chronic conditions reported good health and as few as 19% reported poor health. In respect to income, the highest proportion of respondents (81%) reporting good health was from the high income category and the highest reporting poor health (24%) was from the low income category. Elderly persons who performed no physical activity reported poor health more (79.2%) compared to reporting good health (22.8%). A great number of non-users of tobacco (77%) reported good health while the highest proportion of poor health (26%) was reported by current users of tobacco.

As for regions, the highest proportion of respondents (91%) reporting good health was from the Upper East region and the highest proportion (30%) reporting poor health was from the Western region. All these variables showed a statistically significant difference in SRH except the variables health insurance, locality, alcohol, subjective well-being, quality of life and functioning assessments.

#### Socio-demographic/health related factors and self-reported health

Table 3 shows results of a multivariate logit model explaining the relationship between SRH and the independent variables. From model 1, a statistically significant association can be seen between age and SRH after controlling for all life-course exposures used in this analysis. As age increases by one unit, the odds of reporting poor health against good health is 2.5 times more among the oldest old [OR = 2.5 at 95% CI (1.94–3.35)] compared to the young old. In other word, the odds of reporting poor health increases with age. At 5% level of significance, there was no statistical difference in SRH among the middle old. Respondents who had never worked in a lifetime were two times more likely to report poor health compared to those working in the informal sector [OR = 2.3 at 95% CI (1.60–2.06)]. Similarly, respondents with chronic conditions were 1.6 times [OR = 1.63 at 95% CI (1.33–2.10)] and 2 times [OR = 1.9 at 95% CI (1.41–2.52)] more likely to report poor health if they had one or more than one chronic conditions respectively. The more medical conditions an individual has, the more likely he/she is to report poor health.

**Table 3:** Multivariate model of SRH on sociodemographic/health-related factors

Variable	Model 1^a^ (Binary logistic regression of SRH)		Model 2^b^ (Ordinal logistic regression SRH)	
	Adjusted odds ratio (Conf. inter.)	*P* - values	Adjusted odds ratio (Conf. inter.)	*P* - values
Age				
Young old (60–69)	1.00			
Middle old (70–79)	1.25 (1–1.57)	0.054	1.00 (0.85–1.19)	0.967
Oldest old (80 and above)	2.54 (1.94–3.35)	<0.000	1.01 (0.81–1.26))	0.957
Gender				
Male	1.00			
Female	1.12 (0.86–1.46)	0.402	1.03 (0.84–1.26)	0.792
Marital status				
Married	1.00			
Never married	2.20 (0.93–5.19)	0.071	0.68 (0.32–1.47)	0.327
Divorced	0.95 (0.68–1.32)	0.763	0.81 (0.63–1.04)	0.100
Widowed	1.02(0.77 1.35)	0.885	0.97 (0.79–1.20)	0.803
Education				
No education	1.00			
Primary education	0.83 (0.63–1.11)	0.211	0.97 (0.78–1.20)	0.772
Secondary education	0.83 (0.59–1.17)	0.280	1.18 (0.91–1.52)	0.203
Tertiary education	0.47 (0.21–1.06)	0.069	1.01 (0.62–1.66)	0.954
Work status				
Informal worker	1.00			
Public worker	0.97 (0.64–1.48)	0.894	1.11 (0.83–1.50)	0.468
Private worker	0.93 (0.49–1.76)	0.815	1.22 (0.79–1.91)	0.360
Never worked	2.33 (1.17–4.66)	0.016	1.25 (0.72–2.19)	0.428
Insurance				
Not insured	1.00			
Insured	0.97 (0.78–1.19)	0.747	0.91 (0.78–1.07)	0.254
Chronic condition				
No chronic condition	1.00			
One chronic condition	1.63 (1.30–2.06)	<0.000	0.96 (0.80–1.15)	0.660
More than one condition	1.88 (1.41–2.52)	<0.000	0.99 (0.78–1.26)	0.952
Income quintile				
Low income	1.00			
Middle income	0.93 (0.72–1.21)	0.598	1.06 (0.87–1.30)	0.570
High income	0.71 (0.55–0.91)	0.008	0.89 (0.73–1.08)	0.227
Locality				
Rural	1.00			
Urban	0.98 (0.78–1.23)	0.877	1.12 (0.94–1.33)	0.206
Subjective well-being				
Not Satisfied	1.00			
Satisfied	0.73 (0.59–0.93)	0.009	0.27 (0.22–0.32)	<0.000
Quality of life				
Poor	1.00			
Good	1.02 (0.82–1.26)	0.891	1.03 (0.86–1.22)	0.764
Functional limitation				
Not functionally limited	1.00			
Functionally limited	0.88 (0.67–1.17)	0.379	3.57 (2.88–4.41)	<0.000
Disability				
Not disabled	1.00			
Disabled	0.76 (0.57–1.02)	0.071	2.64 (2.13–3.27)	<0.000
Physical activity				
None	1.00			
Moderate exercise	1.81 (1.37–2.40)	<0.000	3.27 (2.67–4.00)	<0.000
Tobacco use				
Never used	1.00			
Previously used	0.64 (0.45 0.92)	0.017	0.77 (0.61–0.98)	0.033
Currently use	1.19 (0.88–1.62)	0.257	0.87 (0.69–1.11)	0.260
Alcohol use				
Non drinker				
Previous drinker	1.20 (0.94–1.55)	0.150	0.83 (0.68–1.00)	0.053
Current drinker	1.18 (0.92–1.52)	0.201	0.59 (0.49–0.72)	<0.000
Regions				
Upper West				
Western	2.34 (1.14–4.80)	0.021	1.31 (0.79–2.16)	0.299
Central	1.51 (0.73–3.13)	0.268	1.11 (0.67–1.85)	0.673
Ashanti	1.67 (0.82–3.40)	0.160	1.30 (0.79–2.13)	0.294
Brong Ahafo	1.45 (0.69–3.02)	0.325	1.46 (0.88–2.44)	0.140
Greater Accra	1.11 (0.53–2.34)	0.775	1.26 (0.76–2.09)	0.376
Upper East	0.49 (0.21–1.16)	0.103	0.94 (0.55–1.59)	0.813
Northern	1.76 (0.82–3.74)	0.144	1.23 (0.72–2.08)	0.446
Eastern	2.21 (1.08–4.51)	0.029	1.53 (0.93–2.52)	0.090
Volta	1.83 (0.89–3.78)	0.102	1.43 (0.86–2.37)	0.169

^a^Where SRH is coded as follows, very poor/poor = 1, otherwise 0

^b^Where SRH is coded as follows, very poor = 5, otherwise 1–4

Those in the highest income quintiles were 29% [OR = 0.71 at 95% CI (0.59–0.91)] less likely to report poor health compared to those in the lower income quintiles. As income increases, the chance of reporting poor health reduces. Satisfaction with life and surroundings among elderly persons reduces the chances of reporting poor health by about 26% [OR = 0.74 at 95% CI (0.59–0.93)]. Respondents who engaged in mild to moderate exercise were 1.5 times more likely to report poor health compared to their sedentary counterparts. Additionally, previous users of tobacco were 36% less likely to report poor health in contrast to reporting good health [OR = 0.64 (0.45–0.92)].

In comparing the nine regions of Ghana to the Upper West region which is one of the poorest and least developed regions in the country, the odds of reporting poor health was 2 times more among residents in the Western [OR = 2.34 at 95% CI (1.14–4.8)] and Eastern [OR = 2.21 at 95% CI (1.08–4.51)] regions. There was no statistical difference in SRH within other regions. In the same way, there was no significant association between SRH and the following categories; marital status, sex, education, health insurance, quality of life and locality.

In Model 2, elderly persons with functional limitations and disabilities had the odds of 3.6 [OR = 3.57 at 95% CI (2.88–4.41)] and 2.6 [OR = 2.61 at 95% CI (2.13–3.27)] respectively, of reporting very poor health compared to reporting good health. Similarly, current alcohol drinkers were 41% less likely to report very poor health [OR = 0.59 at 95% CI (0.49–0.72)].

## Discussion

The study has analysed factors that influence the elderly self-reporting poor health compared to reporting good health. Age, prior employment, existing chronic conditions, income status, subjective well-being, physical activity, former use of tobacco, functional limitation and disability, current alcohol use and regional residence in Eastern and Western regions of Ghana have been shown to be significantly associated with SRH.

Age is seen here as a significant positive predictor of SRH. Respondents in the old-old category (80 and above) were more likely to report poor health compared to reporting good health. This result is consistent with other studies that have found age to be associated with poor health [47, 48]. The biological process of ageing could possibly explain this correlation; though old people tend to experience decline in mobility and other physical and health conditions, requiring support to undertake usual activities, the situation tends to be worse for older people within the elderly category. Besides, the social (including health) infrastructure in Ghana tends not to be supportive of everyday life and care for older persons – for instance, there are few or no specialist gerontologists in most African countries including the Ghanaian health systems [49]. Thus, this study’s result is expected. A similar study in Thailand found old age to be positively associated with poor health [50] due to decline in mobility and activities. In Japan, Liang et al. found poor health to be highest among those aged 60–85 [51]. According to some authors, increasing stressors and poor conditions increase the chances of disability, frailty and death, thereby outlining the importance of good wellbeing earlier on in life [52]. The cultural setting in Ghana (i.e. the extended family system) provides some form of guaranteed support by their children and grandchildren [53]. However, the current day modernisation and urbanisation of the Ghanaian society has led to economic hardship with more focus on the nuclear family and a collapse of the traditional family support system to elderly persons [54]. This means that reporting poor health could be due to lack of emotional and financial support from loved ones, often feeling that they have become a burden to society.

Previous studies have shown that elderly persons with chronic conditions report poor health compared to those without these conditions [29, 55]. This study showed similar findings. One possible explanation for this is that over 89% of Ghanaians including elderly persons lack in-depth knowledge of diseases and disease preventive practices [56]. Even the National Health Insurance Scheme (NHIS) instituted in 2004 to increase access to healthcare service failed to cover a significant proportion of elderly persons. This invariable implies less access to healthcare services for the elderly, late detection of diseases, rising incidence of chronic diseases and consequent poor health outcome. Equally, the rising incidence of non-communicable diseases (NCDs) can be partly attributed to the increasing ageing population and poor lifestyle [57–59]. High incidence of NCDs indicates increase healthcare needs for the elderly with associated cost implications in the future [60]. Society’s inability to meet these needs likely impacts the quality of life of older populations.

Income was shown to have a negative effect on SRH; Respondents of the highest income quintiles were less likely to report poor health compared to those in the middle and low income quintiles. In other words, low income earners were more likely to report poor health. With higher demand for health care, a population’s affordability of health care services remains crucial in lessening the burden of diseases. This finding is consistent with Tajvar et al. who found that most elderly Iranians who were poor and lived under poor conditions with little or no earnings, perceived their health to be poor [61]. It is known from literature that people in the high income groups can easily afford healthcare and as such, take care of their healthcare needs better and live longer [62]. By implication, high income increase the ability to purchase healthcare services. Also, some scholars found that high income exposes individuals to a good living environment which positively impacts on health [63]. Others hold that low income leads to a feeling of incompleteness, inferiority complex and psychosocial stress [64, 65], which in themselves influence self-reported health status. Some authors carried out a similar study in Ghana with related findings [35]. In the contrary, some authors found no association between income and individual health status, most likely due to the use of an objective measurement of health state [66].

In line with previous studies [67, 68] we found prior unemployment to be related to low health perception. In this analysis, the absence of work invariably increases the chances of reporting poor health in comparison to working in the informal sector were over 70% of Ghanaians obtain their livelihood from. The resultant effect of lifetime joblessness is financial insecurity, otherwise seen as potential risk to poor health. It is important to note here that according to the 2010 population and housing census report, most elderly Ghanaians who were self-employed engaged essentially in agricultural activities (63%), service or sales workers (13.1%) and just a few proportion were professionals (2.7%). Though informal sector workers in Ghana seemed better off when compared to those who have never worked in a life time, low income earnings and lack of social security remains a problem at old age.

In relation to subjective well-being, satisfaction with certain aspects of life was used to assess individual health state. The results showed that persons with high feeling of satisfaction were less likely to report poor health. Supportive findings in Brazil revealed similar association [69, 70]. Elderly people’s satisfaction in Ghana often relates to having children, living with family members and involvement with community affairs [71].

Exercise improves individual fitness, health and general wellbeing. Most studies have found a significant relationship between physical inactivity and poor health [28, 72]. Our study showed the contrary. Respondents who were engage in mild to moderate exercise for 10 min a day were more likely to report poor health. This is rather surprising. Some researchers however argue that few people may actually meet the minimum levels of physical activities which can lead to any health benefit [73, 74]. Others hold that increase stress levels may render physical activities less beneficial [75, 76]. For example, individuals may feel bad being criticized for weight gain or one’s inability to keep up with exercise pace may stir up a feeling of embarrassment and subsequent less pleasure for such activity. These unpleasant life events may alter an individual’s pace of physical activity, cause program attrition and subsequent poor health.

Respondents who previously used tobacco were less likely to report poor health. A plausible explanation could be that early cessation of tobacco reverses harmful effects in the body, improves individual health state and thus lead to a better health outcome [77, 78]. This is contrary to Nakamura et al. [79] who carried out a study on SRH among former smokers in Japan and found poor SRH to be associated with former smokers. According to the authors, bad health may be one of the reasons for quitting smoking in the first place. Such individuals in that case are more likely to report poor health. Contrastingly, in Ghana, the prevalence of tobacco use is low compared to other African countries [80]. The low prevalence could be attributed to the advertising ban on tobacco use in 1982 by the government of Ghana. This ban was later reinforced through the public health act 851 in 2012 were public smoking was completely prohibited [81]. Ever since, it has become increasingly difficult for Ghanaians to smoke in the open consequently increasing the tendency to quit smoking early, thereby reducing the danger of falling into poor health later on in life. In respect to alcohol consumption, the results reveal further that current alcohol consumers are less likely to report poor health contrary to other studies [82, 83]. A major limitation of this finding could be the inability to classify alcohol consumption in terms of content and quantity consumed by respondents. Related studies however, find moderate alcohol beverages to be associated with certain health benefits such as reduction in heart disease [84] and stroke [85].

Regional variations in reporting poor health have been shown to exist from previous studies [86, 87]. Mediating factors may include environments with poor access to health care, low political engagements, lack of transport services and low income generating activities [88]. It is therefore necessary to assess regions with poor health status and determine which areas are a country’s priority to government interventions and better policies. Contrary to the notion of poor health being less in the more affluent cities and regions, we found that elderly persons living in the Western (one of the richest region in the country) and Eastern regions of Ghana, were more likely to report poor health compared to the poorest Upper West region. The Western and Eastern regions second to the Ashanti region contain the highest proportion of elderly persons. These two regions are most suitable for agricultural activities and attract migrants particularly elderly persons engaged in agricultural activities [53]. Internal migrations in Ghana may resort to the elderly living in slums, under poor social and economic conditions and consequent poor health.

Functional limitations and disabilities are risk factors to poor quality of life. Some authors suggest that this relationship is worst in persons with moderate to severe cognitive impairment or with dementia [89–91]. In addition to the wealth of evidence that exists [92–94], our study found a significant relationship between reporting poor health and functional limitation and disability irrespective of cognitive ability. This is in concordance with Depuur et al. who found functional limitation to be associated with poor health among elderly Ghanaians [35]. Most elderly persons in Ghana live in rural areas, in the context of inadequate access to health and other social services, all factors that enhance individuals reporting poor health. Nevertheless, some authors have found this relationship between functional limitation and poor health to be insignificant among blacks though not among whites [95]. The insignificant findings could be related to information bias in which case elderly persons under report their limitations given the sensitivity of the question. We also did not find any significant association between SRH and marital status, health insurance, education, locality and quality of life owing to the confounding effects of other variables.

### Study limitations

A causal relationship cannot be ascertained between the social factors and self-reported health because the study was cross-sectional. However, it is recommended that future research be carried out in form of a cohort study to establish a cause and effect relationship. Further, although previous studies have often used SRH to compare major differences across males and females, rich and poor, or demographic and socioeconomic variables, it has its own drawbacks. Heterogeneity is one of the challenges often faced as individuals may have differences in understanding the question often asked. This commonly causes under reporting or over reporting of health states [96]. Again, SRH may limit people’s experiences and thinking, with women often seen as being sentimental in reporting their health state. However, studies on SRH have been carried out in 70 different countries with good results [97]. Lastly, the use of income as a measure of socioeconomic status was a drawback in the sense that in developing countries, income is often self-reported, and as such respondents may be biased in reporting monthly income in hope of receiving financial aid [98].

### Policy implications

The health status of the elderly is of growing public health concern owing to the social and economic implication of their increasing demographic profile. This study shows that the wealthier enjoy better health compared to the poor. It is therefore important that the health and income security of elderly persons should be of priority to Ghanaian authorities. Government is encouraged to improve economic and social integration through its policies and decisions. For example, employing more youths into the public sector will improve living standards and chances of social security in old age.

The Ghanaian government and its development partners should continue to reinforce policies that will encourage citizens to quit smoking and avoid other risky behaviours. Cessation of smoking can reverse harmful effects in the body and improve health benefits. Measures should be appropriated to reduce the onset of non-communicable diseases through health promotion activities and disease prevention program. Particular interest should be given to the Eastern and Western regions that have one of the highest proportion of elderly persons engaging in agricultural activities. Their welfare and health needs should be prioritized in these industrialised cities to avoid health inequity. Generally speaking, improving elderly health will reduce government expenditure on elderly health needs in future.

Age is already a risk factor for poor health as well as poor performance in basic physical activities. Bearing in mind these risk factors, government is recommended to reinforce its effort to provide a sustainable long term care plan for the elderly and ensure that the existing policies on ageing in Ghana, adopted from the Madrid International Plan of Action on Ageing (MIPAA) be revised and implemented to suit the country’s context. Existing social protection programs such as Livelihood Empowerment against Poverty (LEAP) and National Health Insurance Scheme have helped reduced economic burden and improved health and other aspects, improving living standards. What is needed is sustainability and expansion of such programs to improve the overall well-being of the elderly.

## Conclusion

This study used secondary data from WHO SAGE study in Ghana and used a binary and ordinal logit model to analyse the correlation between SRH and socioeconomic and demographic factors. Self-reported health was found to be significantly related to increasing age, presence of one or more than one chronic condition, physical limitations and disabilities, being a resident of the Eastern and Western regions of Ghana. Also, the results revealed that elderly persons who belonged to the high income quintile, current alcohol drinkers, formerly used tobacco and satisfied with life, were less likely to report poor health. Contrary to other studies, we found mild to moderate physical activity to be positively associated to SRH.

Self-reported health has proven to be a valid indicator in assessing factors that affect elderly health. These factors will increasingly be an issue of concern in Ghana and most developing countries. Managing an aging society in this setting will prove challenging particularly where few health interventions on the elderly exist and geriatric medicine is still an untapped area. It is hoped that this study will pave the way for future studies to explore further, the inexhaustible factors that impede elderly health in Ghana using different research methods. Findings will be used to enlighten policy makers, governing bodies, health professionals and justify the compulsion to implement policies that will help plan for an ageing society. Societal growth is realized when social, economic and health conditions of individuals are improved.

## Abbreviations

ADL: Activities of Daily Living
IADL: Instrumental Activities of Daily Living
MIPAA: Madrid International Plan of Action on Ageing
NCDs: Non-Communicable Diseases
SAGE: Study of Ageing and Adult Health
SRH: Self-Reported Health
